# The Pathogenesis of Tuberculosis–The Koch Phenomenon Reinstated

**DOI:** 10.3390/pathogens9100813

**Published:** 2020-10-04

**Authors:** Robert L. Hunter

**Affiliations:** Department of Pathology and Laboratory Medicine, University of Texas Health Sciences Center at Houston, Houston, TX 77030, USA; Robert.L.Hunter@UTH.TMC.EDU

**Keywords:** Tuberculosis, human, lung, pathogenesis, post-primary, animal model, pathology, X-ray, granuloma, Koch, hypersensitivity, caseation

## Abstract

Research on the pathogenesis of tuberculosis (TB) has been hamstrung for half a century by the paradigm that granulomas are the hallmark of active disease. Human TB, in fact, produces two types of granulomas, neither of which is involved in the development of adult type or post-primary TB. This disease begins as the early lesion; a prolonged subclinical stockpiling of secreted mycobacterial antigens in foamy alveolar macrophages and nearby highly sensitized T cells in preparation for a massive necrotizing hypersensitivity reaction, the Koch Phenomenon, that produces caseous pneumonia that is either coughed out to form cavities or retained to become the focus of post-primary granulomas and fibrocaseous disease. Post-primary TB progresses if the antigens are continuously released and regresses when they are depleted. This revised paradigm is supported by nearly 200 years of research and suggests new approaches and animal models to investigate long standing mysteries of human TB and vaccines that inhibit the early lesion to finally end its transmission.

## 1. Introduction

Improved understanding of the pathogenesis of tuberculosis (TB) tops most lists of needs for developing more effective vaccines and therapies [1]. Today, most pathogenesis research uses sophisticated technologies to investigate the cells, molecules and pathways in animal models. Unfortunately, none of the animal models reproduce the entire disease as it occurs in humans. Especially, none naturally mediate transmission to new hosts and none develop delayed type hypersensitivity (DTH) reactions with the intensity of tuberculous humans [2,3,4]. Investigators in the pre-antibiotic era had materials for research that do not exist today. They observed individual patients or whole families with TB in specialized sanitaria for months or years. They had astute physical examinations, X-rays, skin tests and autopsies of patients with untreated TB. Three-dimensional X-ray and clinical findings were correlated with histologic changes at autopsy. Animal models were developed to specifically reproduce particular aspects of the human disease. A four year study of caseation used at least 200,000 sections of tissues from human autopsy and surgical cases or tuberculous guinea-pigs [5].

**Figure 1:**
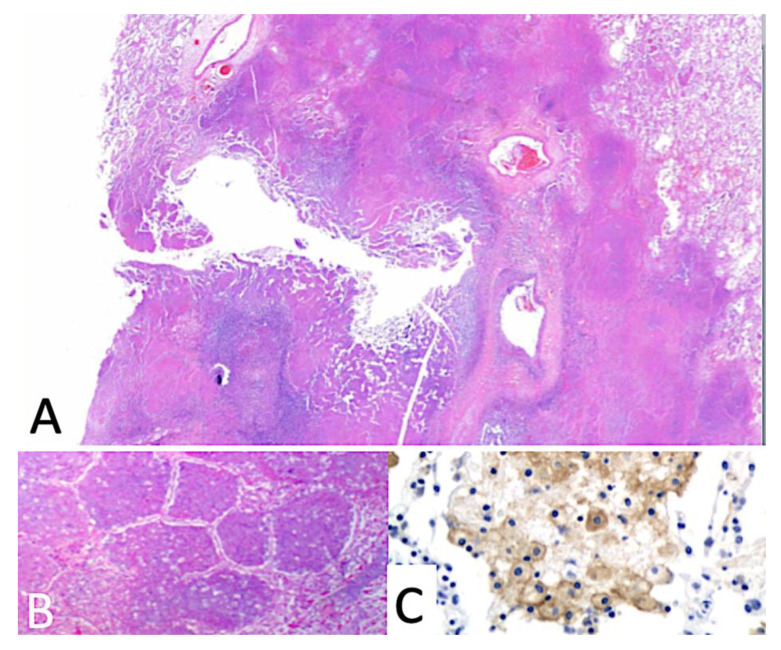
Human cavity formation. (**A**) cavity forming by dissolution of caseous pneumonia. The cavity is lined by softened necrotic lung some of which has already been coughed out. (**B**) Higher magnification of surrounding area showing caseous pneumonia. (**C**) Viable nearby lung with secreted mycobacterial antigens in alveolar macrophages. These antigens with highly sensitized T cells mediate the disease. (A H&E 10×, B H&E 100×, C IHC stain for secreted mycobacterial antigens 400× magnification).

Today, granulomas are almost universally considered the hallmark of TB and cavities are thought to arise by erosion of granulomas into bronchi. This concept arose from studies in animals in the late 20th century and has no support among investigators who studied the pathology of developing post primary TB (PPTB) [6,7,8,9]. Arnold Rich said it well “It has been found by all who have studied early human pulmonary lesions that they represent areas of caseous pneumonia rather than nodular tubercles” [2]. I have reviewed the pathology of over 200 cases of untreated developing PPTB and have confirmed that cavities arise from dissolution of caseous pneumonia frequently in people who have no granulomas in their lungs, Figure 1. The pathology of primary and post-primary TB in humans has been recently reviewed [9]. Granulomas in PPTB arise only late as a reaction to necrotic caseous pneumonia that is not coughed out in formation of cavities. Cavities are a manifestation of the Koch phenomenon, not of granulomas.

In the preantibiotic era, it was widely recognized that the initial infection sensitizes the host so that subsequent infections produce fundamentally different lesions. “The patient’s reactivity, as far as tubercle bacilli are concerned, is never the same after his body has once harbored a tubercle; likewise, the course of the disease, when due to a first infection, is never the same as when due to reinfection” [10]. The first infection of a person produces primary TB that induces immunity to disseminated infection. Any subsequent infection of a sufficiently sensitized host produces PPTB that is restricted to the lung, causes much necrosis and cavities that mediate transmission to new hosts [11]. An enduring mystery is that bronchogenic spread, caseation and cavitation of PPTB are evidence of a considerable degree of immunity to primary TB [12,13,14,15]. Many have been impressed on the one hand by the susceptibility of infants and young children to infection and on the other hand by the difficulty of demonstrating that adults exposed to infection acquire the disease [16]. Adults in endemic areas are typically exposed many times before developing disease and most never do.

Today the research situation has changed completely. Human lung tissue from autopsies is seldom available. Investigators use animal models that do not produce PPTB. They focus on technological advances and study patients only briefly before initiating therapy. The primary granuloma is universally considered to be the hallmark of TB. Investigators of the pre-antibiotic era knew that this is not true. By study of hundreds or thousands of cases they knew that granulomas are not involved in the development of PPTB [2,5,10,11,12,13]. They had a much more nuanced conception of its pathogenesis. Recent studies have identified new components that reinforce their observations and facilitate formulation of more detailed mechanisms. This paper integrates findings from the 19th, 20th and 21st centuries that support a new paradigm for the pathogenesis of TB that restores the Koch Phenomenon as the key driver of PPTB.

Mycobacterium tuberculosis (MTB) is not a typical bacterial pathogen. It is a very successful human parasite that has coevolved with humans for many thousands, if not millions, of years [17]. It can infect many species, but is transmitted in nature only by humans and has no environmental reservoir. Consequently, its survival depends on transmission from person to person. MTB has evolved to avoid destruction by our innate and adaptive immune mechanisms and to induce lesions that facilitate its transmission [18]. It is evolutionarily incentivized not to cause death or disease severe enough to inhibit transmission as this would lead its extinction [14,19].

MTB does not follow the typical rules of bacterial pathogens. WHO estimates that it infects about 1.8 billion people, 25% of the world’s population, and kills more people than any other infection [20]. Yet, over 90% of those infected never get sick. In the 1920s, apical scars or encapsulated apical TB were found in most adult Americans who died from causes other than TB, indicating that spontaneous regression is common and immunity can be highly effective [16]. However, neither immunization, natural infection nor chemotherapy can produce immunity to recurrent adult TB [18]. Dubos wrote, “To have passed through a period of high mortality risk confers not protection, but added hazard in later life” [21]. Humans cured by chemotherapy can become reinfected from the environment within weeks [22]. In addition, there is no correlation between the degree of hypersensitivity and degree of acquired resistance in man. In fact, there is an inverse relationship–the greater the degree of tuberculin hypersensitivity, the greater the susceptibility to disease and death [23,24]. This was confirmed recently in cancer patients treated with checkpoint inhibitors that both enhanced their immune responses and produced reactivation of TB [25]. As stated by Robert North, “A central problem in tuberculosis research is to explain why immunity to infection does not enable mice, guinea pigs, rabbits or susceptible humans to resolve lung infection and thereby stop development of the disease” [18]. Using chemotherapy or vaccines to reduce the MTB load in the lungs by 2 logs does not enable immunity to cause the much lower level of infection to resolve [18].

## 2. The Koch Phenomenon

While adult pulmonary TB is frequently considered to involve pathological manifestations of a hyperactive anti-mycobacterial immune response, key details have been lost [26]. The Koch phenomenon today is largely a historical curiosity that has the potential for causing toxicity of therapeutic vaccines [27,28,29,30,31,32]. In the preantibiotic era, it was considered central to the pathogenesis of TB and responsible for the death of many people. In 1927, Pottenger wrote, “No matter what phase of tuberculosis one may be interested in, whether clinical, laboratory, experimental or immunological, he should familiarize himself with the Koch phenomenon; for in the elaboration and accurate interpretation of this observation lies the understanding of all the reactions which take place between bacillus and host, after infection is once established” [10].

When Koch discovered tuberculin, he regarded it at first as a specific cure for TB. In early attempts at therapy, he studied people with lupus vulgaris (skin TB) because he could watch the lesions. Large doses of tuberculin were injected subcutaneously away from the lesions. A few hours later, TB skin lesions swell, redden and finally become necrotic. Koch reported that tuberculin did not destroy the tubercle bacilli, but only the tuberculous tissue. The inflammation was restricted to the diseased parts only. It did not attack any sound and healthy parts of the body. However, even the smallest otherwise invisible TB lesions were made perceptible through the inflammation [4]. In further studies, human patients proved extraordinarily more sensitive to tuberculin than the guinea and patients with advanced pulmonary TB were far more sensitive than those with lesser tuberculous afflictions [3,4]. It soon became evident that tuberculin therapy was not a cure. The injections induced perifocal reactions and reactivation of tuberculous lesions in each and every part of the body. In the lung, these “tuberculin pneumonias” progressed rapidly to caseation and caused death of many patients [33].

**Figure 2:**
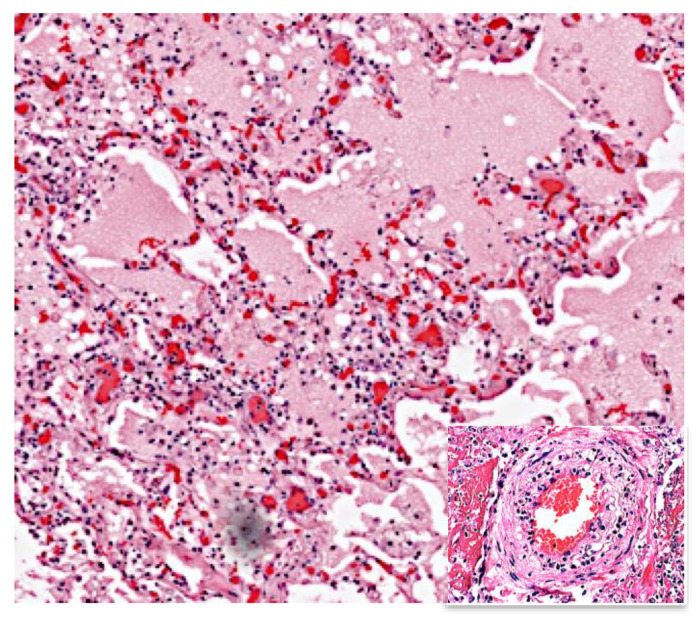
Perifocal Inflammation is a hypersensitivity reaction to tuberculin that surrounds recent, but not old, lesions of both primary tuberculosis (TB) and primary TB (PPTB). It consists of edema and interstitial lymphocytic inflammation. It is a major cause of toxemia and death of adults with acute TB. It is a toxic edema (delayed type hypersensitivity) that usually contains no acid fast organisms. It is typically associated with vasculitis (insert) (H&E stain 100×).

A perifocal reaction is an exudative tissue hypersensitivity reaction that contains few or no tubercle bacilli that surrounds a tuberculous focus, Figure 2. As discussed later, it is probably a manifestation of Type IV or delayed type hypersensitivity (DTH) reaction against secreted tubercle proteins. In the lung, it is composed mainly of alveoli filled with blood plasma, fibrin, red blood cells, relatively few polymorphonuclear leucocytes, many lymphocytes, desquamated alveolar epithelial cells and macrophages. The histological character of a perifocal reaction may vary from purely hemorrhagic perifocal zones to areas of leucocytic infiltrations with marked desquamation and proliferation of alveolar epithelial cells with lymphocytes and plasma, or there may be merely an exudation of plasma with a fibrin and a few lymphocytes [34,35]. They typically contain vasculitis and blood vessels immediately surrounding lesions are thrombosed.

Pottenger wrote, “The reaction which results from the escaping bacilli coming in contact with the immunized cells of the host is not confined to the point of attempted implantation”. Some bacillary substance is set free which acts upon cells in all parts of the body and which may cause an allergic response in foci of previous disease. Not infrequently do we find evidence of this focal allergic reaction manifesting itself as an increased inflammatory activity in foci distant from the point of new invasion; in distant parts of the same lobe, in other lobes of the same lung, in the other lung, or in other organs” [10]. Physical manipulation of tuberculous lesions by collapse therapy or surgery can produce massive perifocal inflammation within 24 h [11,36]. TB progresses if the bacillary antigens are continuously produced and regresses if these antigens are destroyed [37].

Injections of tuberculin cause perifocal reactions in susceptible patients that are indistinguishable from those spontaneously arising [34]. The greater the amount of tuberculin injected, the more intense will be the ensuing perifocal reaction around distant tuberculous foci. This is a well known hazard of skin tests in tuberculous patients. ‘It is particularly in the recent and in the unstabilized tuberculous lesions that we must beware of the potential dangers of the Mantoux test. A well-stabilized lesion, even an extensive one, is much less likely to react unfavorably to a tuberculin test than an unstabilized lesion, although the latter may be small in extent’ [34]. Caseous lesions, especially those without fibrous encapsulation, may show extensive perifocal reactions after tuberculin is injected. The perifocal inflammatory exudate may then undergo caseation and cause progression of the lesion [34]. The tissues surrounding a well-encapsulated or well calcified lesion are less sensitive to tuberculin, and because of this fact a significant perifocal reaction may be absent even after the injection of large doses of tuberculin [34]. However, it is not rare that upper lobe calcified nodules become reactivated with perifocal inflammation, especially in adolescents and produce rapid tissue destruction [11].

Perifocal reactions may surround both primary and PPTB lesions and have variable courses. The age of the subject and size of tuberculin skin test are important [38]. The larger the skin test, the more likely a severe perifocal reaction will develop. TB of the very young is in general without very extensive perifocal inflammation. The complication is found more regularly between the ages of 25 and 30 years. It attains extreme degrees at approximately 40 years and diminishes in importance in the older age group. When patients with perifocal infiltrations on X-ray are put at rest, large areas of infiltration may disappear quickly and recovery is rapid [39]. The lesions also clear rapidly with antibiotics [36].

## 3. X-rays of Developing TB

**Figure 3:**
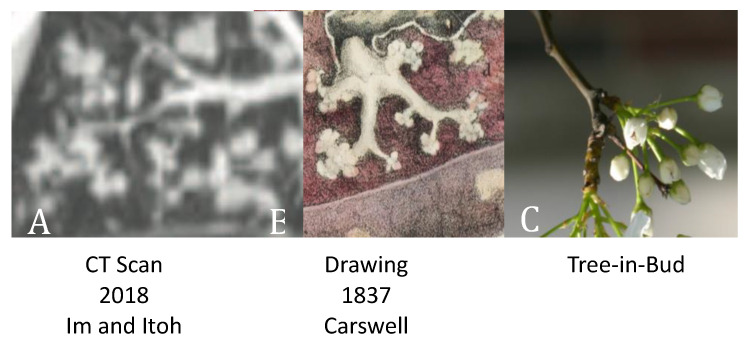
Tree-in-bud sign on CT scans was recently rediscovered as the characteristic early lesion of PPTB. It was described by Carswell in 1837 and by X-rays in the 1920s [42,46,50]. Today, it is recognized as characteristic of advancing post-primary TB.

Recent investigators have proposed that the classification of TB be expanded to include incipient and subclinical TB in addition to active and latent infection [40,41]. This is based in part on a high-resolution CT scan that is the method of choice to reveal the tree-in-bud sign of subclinical TB [42,43]. This sign shows 2 to 4 mm centrilobular nodules and sharply marginated linear branching opacities around terminal and respiratory bronchioles in a pattern that mimics the branching pattern of a budding tree, Figure 3 [44]. Far from being new, this was recognized by multiple investigators a century ago who described it with the terms “studding of bronchi”, “budding twigs,” “raisins on a stem” or “pussy willows.” [45,46,47]. While investigators in the pre-antibiotic era lacked technologies we have today, they had capabilities and resources that no longer exist. The routine followed in Dunham’s studies was to remove the lungs from the body, inflate them to their normal size and make stereoscopic X-rays that were compared with histologic lesions and stereoscopic X-ray studies of the living patient [46]. This made it possible for X-rays to identify in the lungs of children and adults the various lesions of subclinical and active TB that are recognizable at autopsy [48]. Several generations of pathologists and clinicians became familiar with the bronchial spread of the infection through the lungs that led to tuberculous pneumonia, cavitation, post-primary granulomas and fibrocaseous disease as well as the form of its source as ‘budding twigs’ [49]. Most of this has now been forgotten and replaced by the fantasy that granulomas are the hallmark of all TB.

The differences between the X-rays of tuberculous children and adults were described shortly after introduction of X-rays into medical practice [46]. Lesions that were incipient to clinicians were already progressive to the radiologist [45]. The majority of incipient adult lesions were only found by X-raying apparently healthy persons, particularly among groups exposed to massive infection or circumstances lowering resistance. These were not rare. Of 3000 unselected necropsies at Stanford University in 1923, 1905 (65%) had TB [51]. This was far beyond the incidence of any other disease. Of these, there were 525 (29%) active cases of pulmonary TB and 1255 (71%) cases with healed, mostly apical lesions. Among the active cases the mortality was greatest between ages 20 and 40. Opie reported that in Philadelphia, 70% of teen age children of a parent with TB and 40% of such children whose parents were well had pulmonary TB lesions on X-ray and that he could tell months ahead of time which of these would develop clinical disease [48,52]. Early minimal lesions were always unstable, most regressed with rest therapy while some progressed [11].

**Figure 4:**
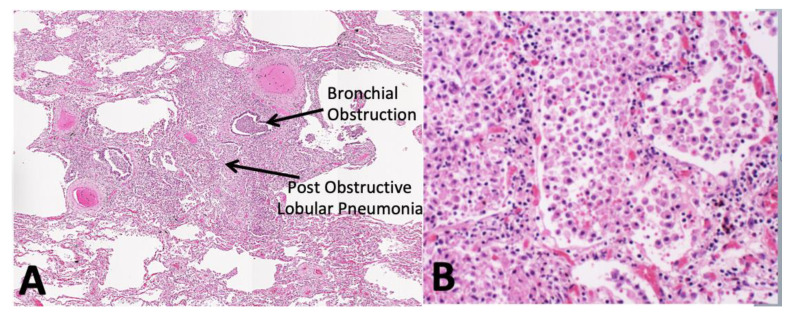
The Early Lesion of PPTB begins as an alveolitis with foamy alveolar macrophages behind an obstructed bronchus (upper arrow) (**A**). Over a period of months, the macrophages sequester mycobacterial antigens and host lipids, while the alveolar walls thicken with lymphocytes to produce obstructive lobular pneumonia (lower arrow and (**B**)). (H&E, A 10×, B 40×).

In 1925, Assmann drew attention to a solitary infraclavicular opacity which he had observed in young adults with slight symptoms, no physical signs and a history of contact with TB. He suggested that this opacity might represent the early tuberculous focus in adults. One could frequently demonstrate that they were TB by culture of gastric aspirates. Subsequent development of open TB in many of his patients substantiated this view [53]. The early pulmonary infiltrate, Assmann’s focus, is the lesion that represents the onset of PPTB, Figure 4 [54]. Worldwide interest in its significance stimulated numerous studies and publications through the 1940s and beyond [43,44,46,48,52,53,54,55,56,57,58,59]. Multiple investigations confirmed that the early pulmonary infiltrate was a common onset of TB. Investigators were able to longitudinally observe the progression and/or regression of subclinical PPTB for months before the onset of symptoms. This stimulated the widespread use of X-rays to detect early TB [54].

Studies of correlation of X-rays of the early lesion with pathological changes in the lung were conducted by multiple investigators [58,59]. The early infiltrates were shown to be small areas of exudative bronchopneumonic TB typically near the pleural surface in the upper posterior part of the lung. Few tubercle bacilli were seen by AFB staining. Using serial X-rays, it was noted that such lesions frequently resorbed completely [11]. The lesions were shown to be fan or wedge shaped centered on a bronchus and extending to the pleura. Pathologically, this wedged shaped TB pneumonia was associated with obstruction of the bronchus and extended to include all of the lung tissue supplied by that bronchus. Surgical relief of such obstruction frequently caused healing [2]. The discharge of bacilli into the sputum might be only intermittent because the semisolid caseous material produced bronchial obstruction that trapped the organisms [54]. Tuberculous bronchopneumonia may exist in a large area without causing any signs or symptoms. Many reach maximum density in a few months and then resolve leaving an apical scar [60]. However, its recognition suggests a grave prognosis. By use of stereoscopic X-rays, the most vital developmental phase of the disease could readily be followed and studied in the living human [46].

Perifocal inflammation is often severe and may cause of death. It is most common in the early phases of tuberculous lesions in patients with strong tuberculin skin test reactions [38]. The larger the skin test reaction or IGRA response of an individual, the greater the tendency to apical localization, perifocal inflammation and death [2,24]. Amberson reported that perifocal haziness on X-ray is an invariable accompaniment of disease found in serial roentgenograms to be progressive [47]. The more intense and the more extensive the perifocal haziness in the X-ray, the sicker the patient is likely to be and the more profound the general intoxication. Perifocal haziness is usually most prominent about bronchopneumonic or pneumonic deposits and, in such circumstances, the patient is usually seriously ill [47]. The disappearance of perifocal haziness is always the first stage of repair and the subsidence of constitutional symptoms of intoxication [47]. The comparison between certain non-tuberculous miliary reactions (silicosis, Boeck’s sarcoid) and acute miliary TB is instructive. The first are well tolerated because of the absence of perifocal inflammation, while the tuberculous infection may be fatal because of a diffuse perifocal inflammation. Perifocal inflammation is the best evidence of the activity of a focus [38].

## 4. Delayed Type Hypersensitivity (DTH)–Both Protection and Perifocal Inflammation

The tuberculin skin reaction, a localized immune reaction to soluble MTB proteins, is the classic model of DTH (Type IV hypersensitivity reaction) and host resistance to TB. Mycobacterial antigens drive the differentiation of CD4^+^ T cells to Th1 cells. Th1 cells secrete IFN-γ that is responsible for macrophage activation as M1 cells that control the infection [61]. However, DTH is capable of much more [62]. The pathology of DTH was studied intensively by Harold Dvorak who used multiple models [63]. He reported that the effector mechanisms of DTH included the coagulation and microvascular systems in addition to macrophage activation. DTH increased microvascular permeability, caused edema, vasculitis, and activation of the clotting system with extravascular fibrin deposition, thrombosis and systemic coagulopathy in addition to macrophage activation and lymphocyte infiltration around blood vessels [64]. These are all features of the perifocal reaction to TB in the human lung; see Figure 2. The deposition of fibrin can account for the gel consistency of some perifocal lesions [65]. In addition, a large Russian series confirmed that patients with acute pulmonary TB have a profound coagulopathy that appears to be caused by procoagulant produced by macrophages under the influence of sensitized T cells [64,66,67,68,69].

Dannenberg and others agreed that DTH was important, but had a much more nuanced conception of its activity than just activating macrophages to kill ingested organisms [70]. He wrote that the pathological features of TB appear to be determined by the interactions between tissue hypersensitivity and local mycobacterial antigen load. With insufficient hypersensitivity, infected macrophages are drawn into granulomas where they die and add to the growing caseum. Where tissue hypersensitivity is high and antigen load sparse, well-formed granulomas with activated macrophages kill MTB and contain infection. When both the antigen load and hypersensitivity are high, the result is a perifocal reaction with massive necrosis that leads to cavitation. Thus, the formerly beneficial DTH reaction that is responsible activation of macrophages to kill MTB is, with excess bacillary antigen, also responsible for almost all of the tissue damage produced by this disease, including granulomas, perifocal reactions, caseation, liquefaction, and tuberculous pneumonia [71,72].

## 5. Early Lesion of Post-Primary TB (PPTB)

There is a disconnect between the current idea that granulomas are the hallmark of TB and earlier observations that ‘Granulomas do not play a role in the development of phthisis in the adult’ [2,11]. The distinctions between the lesions of primary TB and phthisis (PPTB) were recognized grossly by Laennec [73], microscopically by Virchow [74], immunologically by Koch [3], clinically by Osler [75], radiologically by Dunham [46], genetically by Alcais [76] and confirmed by many investigations over nearly two centuries [8,9]. Primary TB produces granulomas are widely studied. PPTB begins as an infection of alveolar macrophages in people with sufficient immunity to heal caseating granulomas [7,77]. It progresses as an asymptomatic obstructive lobular pneumonia, tree-in-bud sign, that spreads via bronchi before undergoing caseous necrosis that is either coughed out to form cavities or retained to become a focus of post-primary granulomas and fibrocaseous disease.

Investigators in the preantibiotic era recognized that caseation necrosis contained much lipid and was due to hypersensitivity, but they could not identify the sources of lipid or antigen. Since infected necrotic material was spread through bronchi, they assumed that the offending antigens were discharged from small necrotic foci into bronchi [2,78]. Recent studies have clarified the situation and led to the present conception of the pathogenesis of TB as described next.

### 5.1. Role of Bronchial Obstruction in Cavitation

It has long been known that cholesterol rich lipids accumulate in alveolar macrophages for months before onset of necrosis to produce caseous pneumonia [5,79,80]. In addition, Osler and others reported that bronchial obstruction is found in 100% of cases of adult pulmonary TB [2,75,81,82]. Furthermore, surgical relief of obstruction has been reported to cause healing of tuberculous infiltrates [2]. We now know that bronchial obstruction by foreign bodies or tumors causes lipids to accumulate in post-obstructive lipid pneumonia that has a propensity to cavitate [83,84]. It is known as golden pneumonia because of the yellow color of large amounts of lipid. The lipid derives from pulmonary surfactant that is degraded and stored in foamy alveolar macrophages.

We reported a case of a cavity in a patient with cancer that has many similarities to those produced by TB [85]. Obstructive lipid pneumonia formed behind a bronchus obstructed by cancer. Chemotherapy caused necrosis of the lipid pneumonia that was coughed out to produce a large cavity. The man died a few days later. Histologically, the cavity contained areas of lipid accumulation in necrotic foamy macrophages within alveoli, a fibrinous exudate and lymphocyte infiltration of alveolar walls. There were even a few giant cells that resembled Langhans giant cells. The walls of the cavity resembled those of developing cavities of TB. The necrotic material in the wall was lipid rich necrosis resembling caseation. This case is not unique. Cavitation has been reported in a significant proportion of cases of obstructive lipid pneumonia suggesting that it is a factor in development of cavities in PPTB [35,85].

Bronchial obstruction is not the only factor that impedes clearance of materials from bronchi in the early lesion of PPTB. MTB promotes dysregulated lipid metabolism in macrophages that promotes foam-cell formation [86]. In addition, the upper lobe has the lowest, movement, ventilation, perfusion and lymphatic flow of any part of the lung [35,87,88]. Ahoen reported abnormalities of bronchial cilia in pulmonary TB that would greatly impair their function [89]. The importance of alveolar clearance by cilia in the defense against TB is supported by the effects of cigarette smoke that paralyzes cilia and is a significant risk factor for clinical PPTB [90,91]. Another factor may be damage to nerves supplying cavities that may further impede motion [85]. Finally, alveolar macrophages in normal individuals carry MTB into the lung interstitium where they establish granulomas. This stops with the development of hypersensitivity. MTB infected alveolar macrophages then remain in alveoli where they become foamy due to accumulation of lipids. All of this provides an environment where alveolar macrophages can be isolated and sequestered for months slowly developing conditions for caseation pneumonia. Many lesions heal prior to caseation leaving an apical scar (Simons foci) [92]. A few undergo caseous necrosis and are coughed out to form a cavity or remain to become the focus of post-primary granulomas and fibrocaseous disease [78].

### 5.2. Synthesis of Secreted Mycobacterial Antigens in Alveolar Macrophages

**Figure 5:**
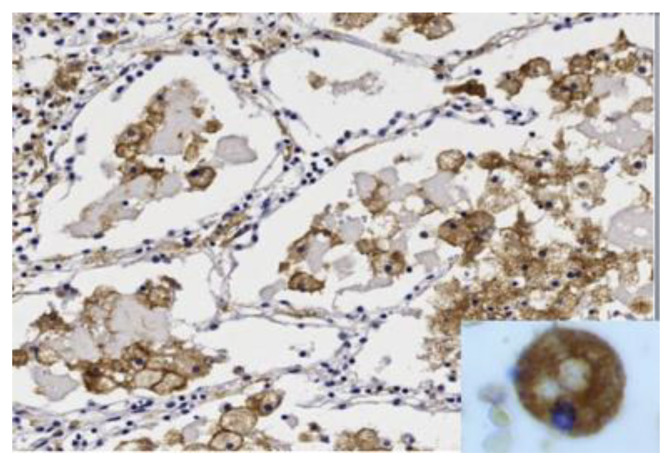
Sequestration of secreted mycobacterial antigens. Secreted mycobacterial antigens are asymptomatically produced and sequestered in foamy alveolar macrophages of the early lesion of PPTB for months before the onset of caseous pneumonia and clinical disease. Simultaneously, the tissue develops extreme hypersensitivity related to T cells in the alveolar walls. Evidence suggests that this is the essential developmental lesion of PPTB. Release of this antigen causes inflammation and necrosis. (Immunostain for mycobacterium tuberculosis (MTB) antigens 400×, Insert 1000×).

Investigators of the early 20th century recognized that the massive necrosis of PPTB is caused by hypersensitivity to mycobacterial antigens. Since all non-necrotic lesions in immunocompetent hosts were paucibacillary, the only sources of MTB organisms and antigens that they could identify were small necrotic foci of lobular pneumonia and ulcerating hilar lymph nodes. Multiple investigators proposed that these lesions seeded bronchi to produce spreading bronchogenic disease with the tree-in-bud pattern [2,49,78,93]. Today, with immunohistochemistry, we can easily identify the source of mycobacterial antigen, Figure 5. It is found in large amounts in innocent looking, foamy alveolar macrophages of the early lesion of PPTB. The early lesion is an obstructive lobular pneumonia with no necrosis and little inflammation that does not begin until after the host has developed significant hypersensitivity [78]. Most clinical TB today in endemic areas is from recently transmitted infections [94,95]. This implies that PPTB can begin from newly inhaled organisms in addition to existing small cavities or reactivation of dormant bacilli. Once started, the infection spreads via bronchi throughout an entire lobe to produce the wedge or fan pattern on X-ray.

### 5.3. TDM as an Invisibility Cloak for Intracellular MTB

PPTB begins with prolonged asymptomatic accumulation of secreted mycobacterial antigens in alveolar macrophages in highly sensitized tissue of the early lesion. It accomplishes this with little or no inflammation even though the antigens reside in highly sensitized tissue and there may be intense tuberculous inflammation to the same antigens elsewhere in the same lung [35]. We published evidence that trehalose 6,6′ dimycolate (TDM) or cord factor forms an inert covering of MTB (an invisibility cloak) that may contribute to its ability to persist in such lesions without producing inflammation [96].

TDM is the most abundant lipid produced by MTB. It is found free on the surface of MTB and is responsible for the formation of serpentine cords that are an ‘essential accompiant of virulence’ [97,98,99]. With fatty acid chains of over 70 carbons, TDM is totally insoluble. The fact that MTB is an obligate human parasite that expends significant resources on its synthesis strongly suggests that TDM is essential for its survival. TDM is unique in that it has three distinct sets of biologic activities depending on its physical conformation [100,101,102,103,104]. As a single molecule, TDM stimulates macrophage C-type lectin receptors including Mincle. It can also form three crystal-like structures, cylindrical micelles, intercalated bilayer and monolayer, that have distinct biologic activities. In the monolayer form on lipid, TDM is highly toxic. It destroys cells in minutes upon contact and induces granulomas [105].

TDM free on the surface of MTB exists as cylindrical micelles or an intercalated bilayer that is necessary for survival of the organisms in macrophages and in mice [106,107,108]. Removal of TDM reduced and adding it back restored survival of MTB in both. In its nontoxic form, TDM inhibits phagosome/lysosome (P/L) fusion in macrophages [107,108]. TDM on MTB was found to inhibit multiple other functions as well. It suppressed the ability of viable MTB to stimulate the macrophage surface antigens MHCII, CD1d, CD40, CD80 and CD86, inhibited stimulation of the cytokines IL-12, IL-6, TNF-α and IL-12. TDM on the surface of MTB also suppressed antigen presentation [100,109].

A clue to how TDM on the surface of MTB is able to inhibit so many activities of MTB is the ability of trehalose to simulate water and protect organisms from drying or freezing [96]. The cylindrical micelles and an intercalated bilayer of TDM that protect MTB from killing in macrophages have surfaces composed of tightly packed, rigidly immobilized trehalose. Such trehalose surfaces simulate and bind water far more effectively than free trehalose. We speculated that this barrier of immobilized water constitutes an ‘invisibility cloak’ that facilitates the persistence of MTB in multiple cell types without producing inflammation, even in highly immune individuals [96]. Receptors in or on cells would see only immobilized water rather than any of the ligands present on MTB. This would provide the organisms with ability to expose only ligands of its choosing and thereby manipulate the host’s cells otherwise impossible ways.

### 5.4. Nature of Hypersensitive Tissue–Trm, PD1/PD-L1 and more

Human tissue around foci of developing PPTB is extremely sensitive to tuberculin even when injected at a distant site. Highly sensitive humans react to 0.1 mL of a 1/1,000,000 dilution of old tuberculin that is far smaller than the amount required in sensitized guinea pigs or rabbits [2]. Thus, people can develop positive reactions to a billionth of a milligram of PPD as a result of immunization by multiple asymptomatic infections [110]. These are also the people who experience the greatest development of the early lesion of PPTB and the greatest risk of clinical TB and death. Their tissues are asymptomatically synthesizing and storing secreted mycobacterial antigens in close approximation with highly sensitized T cells. This raises multiple new questions about the nature of sensitized tissue and what prevents the antigen from stimulating inflammatory reactions at the site of its storage.

There is growing understanding that pathogen specific T-cell immunity can be localized at the site of infection due to the existence of tissue resident memory T-cells (Trm) marked by CD69 and CD103 [111]. These cells do not recirculate in the blood and thus do not contribute to studies of the systemic immunity [112]. However, they are ideally situated to mediate a local hypersensitive response in PPTB lesions, the Koch phenomenon [113].

Driven largely by immuno-oncology, much has been learned about the role of PD1/PD-L1 and other mechanisms of immune regulation in tissues [114]. Sections of characteristic lesions of human TB were selected for quantitative immunohistochemical studies [115]. Abundant mycobacterial antigen, but very few AFB, were present in foamy alveolar macrophages of early lesions. Primary granulomas contained a preponderance of CD4^+^ T cells while the early lesions contained more CD8^+^ T cells. In addition, PD-L1 was highly expressed in foamy macrophages, surrounded by PD-1 expressing lymphocytes in the alveolar walls of the early lesion of PPTB [116]. A marker of M2 macrophages, CD163 was found in the same alveolar macrophages as MTB antigen and PD-L1 in developing PPTB. In another study, tissue-resident memory T cell *(*Trm*)* markers, CD 69 and CD103, were found on PD-1 expressing T cells of surgical resections of pulmonary TB [117]. Markers of mTOR signaling (pmTOR, insulin-like growth factor-1 receptor and activated Akt) and a second pathway of macrophage activation, COX-2 [116] were assessed in early lesions of PPTB [116]. The results suggested that foamy macrophages in early lesions over activate mTORC1, potentially inhibiting autophagy of infected cells. Thus, in this critical microenvironment of the early lesion of PPTB, PD1, PD-L1 and two suppressor host response pathways (mTOR and COX-2) appear active on Trm cells. Further studies of the nature of localized hypersensitive tissue and the antigen it contains are needed.

## 6. Later Lesions of PPTB

### 6.1. Primary and Post-Primary Granulomas

**Figure 6:**
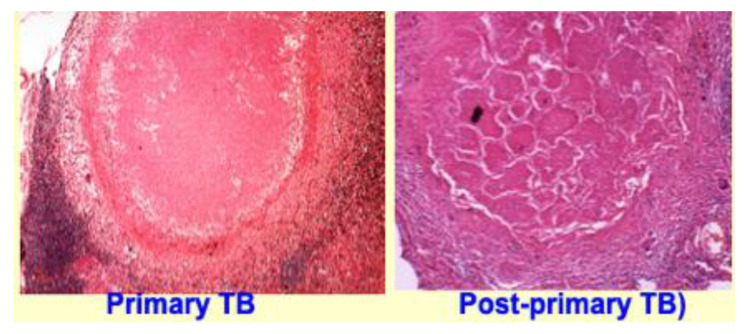
Granulomas of primary TB have homogeneous caseum with peripheral lipids. Granulomas of PPTB consist of ghosts of alveoli (light-colored cracks are alveolar walls) surrounded by granuloma. They are found naturally only in human lungs and form to encase to unexpelled caseous pneumonia [18]. (H&E stain 4×).

Primary granulomas form in individuals whose macrophages are unable to efficiently kill the ingested organisms, Figure 6 [37]. Consequently, lipid rich foamy macrophages are continuously recruited into developing granulomas where they die and contribute to the growing caseation. Within the caseous tubercle itself, no elements of normal tissue are found. The normal tissue is pushed away as the granuloma grows [11]. This typically produces spherical granulomas [118]. This process continues until the epitheloid macrophages gain ability to kill entering organisms and halt growth of the granuloma. Live MTB may persist within the granuloma for years before they die and the lesion calcifies.

Post-primary granulomas develop quite differently. They start as the early lesion of PPTB that undergoes caseation to produce caseous pneumonia [9,77]. Some material is coughed out to form cavities. That which is not coughed out is retained to become the focus of post primary granulomas that produce the nodular reverse halo sign on CT [119]. These lesions are not round, but follow the branching cylindrical shape of the bronchogenic caseous pneumonia that they surround. In describing PPTB, Canetti wrote: “Generally tubercle formation is observed at the periphery of the caseum, and does not precede caseation but follows it. A caseum without tubercles at the border is often seen, but one hardly ever sees tubercles without some neighboring caseum. Moreover, caseum associated with the tubercle is always old and is free of nuclear debris. These two facts establish that the caseum precedes the tubercle” [38]. Since post-primary granulomas surround preexisting foci of caseous pneumonia, they do not grow as do primary granulomas. Medlar who had personally studied thousands of cases wrote “Large numbers, perhaps thousands, of epitheloid tubercles have been studied to determine whether any evidence can be found to indicate a considerable increase in tubercle bacilli and whether an enlarging necrotic lesion might result from a growing tubercle. … In no instance were groups of bacilli found which would suggest either active intracellular or extracellular multiplication. … In no instance was evidence found which would support the proposition that a tubercle was growing” [49].

### 6.2. Non-Cultivable MTB and Their Resuscitation

As a human parasite, MTB has coevolved with us for a very long time [17]. Consequently, we should expect its gene expression to adapt to conditions in our bodies to ensure its survival [18]. MTB must grow rapidly in a cavity to be expelled to infect new hosts. However, if it divides as rapidly in any other part of the body, it endangers its host and itself. A person may survive tuberculous meningitis, but the organisms never do. There are several examples of MTB modifying its behavior in different circumstances of infection [41]. We reported that MTB from pulmonary sites (cavities) grows faster and produces more TDM than the same strains isolated from extra pulmonary sites (granulomas) [120]. Similarly, Rich reported that one cannot predict how long it will take for culture of MTB from extra pulmonary sites [2].

An intriguing example takes place in the lung with the onset of caseous pneumonia. As the early lesion evolves towards caseation there is a veritable eruption of bacilli so that the onset of caseation is associated with a massive increase in the number of acid fast bacilli [38]. However, few or none such bacilli in closed lesions of tuberculous pneumonia will grow in routine culture [38]. This is especially interesting since virtually all of the bacilli in nearby lesions that open into airways will grow rapidly in culture. Similar non-cultivable MTB have been found in sputum samples of sputum culture-negative, smear-negative individuals [41]. Some could be resuscitated by exposure to liquid media supplemented with fresh MTB culture filtrates. The resuscitation-promoting factors are required for virulence and resuscitation from dormancy but are collectively dispensable for growth in vitro [121].

It is interesting to speculate on the role of non-cultivable organisms in the survival of MTB. Very few MTB are present in the early lesion of PPTB, but they are actively producing secreted mycobacterial antigens in preparation for a necrotizing pneumonic reaction sufficient to form a cavity. Most of these will be coughed out with necrotic lung as the cavity forms. MTB thus has a problem. If it divides freely and escapes from developing necrosis, it will endanger its host. If it fails to divide, it risks leaving insufficient organisms to maintain the cavity. The solution is to divide in massive numbers to ensure that adequate organisms remain, but prevent them from dividing until they sense appropriate conditions on the cavity wall.

## 7. Need for a Paradigm Shift

A paradigm is a ‘conceptual world view’ that, for a time, determines the kinds of experiments scientists perform, the types of questions they ask, and the problems they consider important [122]. The National Research Council published a monograph “A New Biology for the 21st Century” that explains that challenge of advancing from identifying parts, to defining complex systems is still well beyond current capabilities [123]. Without an appropriate paradigm, biology ‘hits the wall of biocomplexity’ [124]. This is the state of much current TB research. Today’s prevailing paradigm that primary granulomas are the hallmark of TB arose after closure of TB hospitals when interest had shifted from the disease in humans to studies of the immune stimulating activities of components of mycobacteria in animal models. When interest in TB resumed in the 1980s, investigators simply continued with their animal models that made granulomas since they had no experience with and little access to the actual human disease. Consequently, for the past five decades, modern science has tried to understand the pathogenesis of TB in animals that do not develop the human disease guided by a badly flawed paradigm. While such research has made previously unimaginable progress in defining the parts (the cells, molecules and pathways of TB), it has not been able to put them together into a coherent explanation of the pathogenesis of the disease. This is usually expressed as a need for ‘correlates of immunity’.

Most paradigm shifts are vigorously opposed [122]. Experimental data rarely challenge established paradigms and observations that do not fit are typically ignored. Instead, concepts must evolve and then experiments must investigate these hypotheses [125,126]. The essence of the New Biology, as defined by the National Research Council, is integration—integrating knowledge from many disciplines to permit deeper understanding of biological systems [123]. Fortunately, for us most of the work of integrating had already been done decades ago [2,49]. In 1927, Pottenger wrote that the Koch phenomenon was the key driver of TB disease [10]. In 1947, E.M. Medlar, Chief Pathologist, Division of Tuberculosis of New York State, wrote *“several generations of pathologist and clinicians have been familiar with such important phenomena as the bronchial spread of the infection through the lungs and the form of lesion acting as its source”* [49]. While modern science has gained much from new technologies, it has also lost much because the prevailing paradigm does not recognize the importance of the Koch phenomenon or existence of the bronchial spread of the infection through the lungs or the form of lesion acting as its source.

## 8. Animal Models

The central message of this paper is that the Koch phenomenon (tissue damage due to tuberculin hypersensitivity) is central to the pathogenesis of PPTB. It is important to remember that MTB is an obligate human parasite because no animal can naturally produce lesions like humans that mediate transmission. However, several animals can be induced to produce components of the human disease for study. Unfortunately, animal models in use today are seldom designed to produce or evaluate such lesions. The term post-primary TB refers to the disease that develops in humans following sensitization by primary TB. Typically, such sensitization in humans is the result of multiple subclinical infections. Most animal studies today use only a single infection, frequently a low-dose aerosol exposure. Not surprisingly, this produces only primary TB. Some develop similarities to PPTB, but they are usually ignored. In addition, bacterial burden has been the gold standard to assess disease in animals. This is unfortunate since there is no clear correlation between the disease severity in immunocompetent humans and the numbers of bacteria present [2]. This should be expected in a disease due to hypersensitivity.

Much evidence shows that the differences between childhood and adult TB are due to prior sensitization of the adult. Childhood and adult TB resemble the reactions of non-sensitized and sensitized animals respectively [46,58]. In 1922, Opie wrote “When tubercle bacilli are introduced into a susceptible animal, a lesion is formed at the site of inoculation and dissemination occurs by way of the lymphatics and bloodstream. This lesion, even if the lung is the primary site of inoculation, has little resemblance to the phthisis of adults. If, however, the resistance of the animal is increased by preceding infection, the modified lesion of the lung often presents a close resemblance to human phthisis” [16]. Several recent investigators have recognized the problem. “We really need to generate a better understanding of human TB and identify which aspects can most usefully be modeled in experimental animals. The route to better animal models is inextricably linked to a better understanding from direct studies of human infection” [127]. TB research should be an iterative process with improved understanding of human TB leading to the improved animal models [128]. This happened regularly in the preantibiotic era when human tuberculous tissues were plentiful, but, unfortunately, it seldom happens today because of the decline in interest in and availability of autopsies.

Several investigators reported that the principle features of early pulmonary TB in adult humans, including limitation of the process to the lung, pneumonic and cavitary lesions could be reproduced in rabbits by prior immunization [129,130]. Medlar spent 25 years and employed many artifices in pursuit of ways to replicate all features of the human TB in animals [49]. “Although progressive pulmonary tuberculosis, necrotic lesions, and an allergic state were easily demonstrated, other features commonly found in human pulmonary disease proved difficult to produce.” More recent publications report that sensitized rabbits can develop rapid onset exudative lesions reminiscent of PPTB and granulomas and cavities that develop from alveolitis in a fashion reminiscent to those of PPTB [131,132]. This alveolitis can rapidly progress to pulmonary cavities that share many characteristics with the human lesions [133].

Several species of animal develop chronic TB disease that has some characteristics of the early lesion of PPTB. In mice, guinea pigs, and rabbits, immunity succeeds in inhibiting MTB growth and stabilizing infection at a low stationary level beginning around 20 days after infection [18]. Progressive pulmonary TB in these animals is not due to increasing numbers of viable bacilli, but to a continuous host response to mycobacterial antigens [37]. According to North, ‘A central problem in TB research is to explain why immunity to infection does not enable these animals or susceptible humans to resolve lung infection and thereby stop development of the disease’ [18].

A key point is that most immunocompetent humans do resolve the early lesions of PPTB while most animals eventfully die from them. A perplexing mystery is how strong immunity to primary TB is actually required to initiate PPTB and why people with the strongest skin test reactions are at highest risk of clinical disease and death from it [2,24]. Most adults require multiple exposures to MTB before developing clinical disease perhaps because it takes multiple exposures to develop strong enough immune responses to promote PPTB [134]. It seems that the early lesion of PPTB actually requires a strong immune response to be able to synthesize and store mycobacterial antigens in preparation for a necrotizing hypersensitivity reaction large enough to form a cavity that can transmit infection. Learning why most such lesions resolve spontaneously in humans, might suggest ways to make them all regress and thereby drive MTB to extinction.

**Figure 7:**
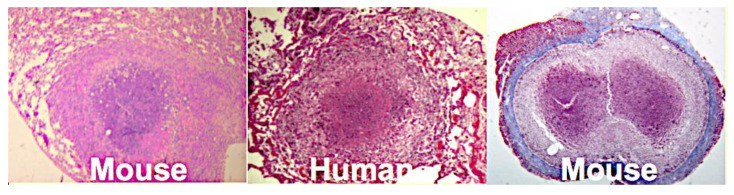
Caseating granulomas in humans and mice. Contrary to widespread belief, mice are able to develop several types of caseating granulomas each of which resembles a type of human lesion. This requires that one duplicate the conditions that occur in humans when the lesions develop rather than the route and dose of infection [135] (H&E stain Left and center) (Connective tissue stain, Right, showing a fibrous capsule).

Mice are widely criticized as a model of TB because they do not produce caseating granulomas following single infection with MTB. However, we produced classic caseating granulomas in mice by reproducing the conditions in them that exist in humans during the development of such lesions [135]. This involved injection of MTB or trehalose 6,6′ dimycolate (TDM) in oil emulsions into sensitized mice, Figure 7. Two mechanisms of necrosis were identified in such lesions. The first was a T cell reaction specific for TDM. The second appeared to be infarction produced by vascular occlusion as observed in DTH. By modification of the immunization protocols, dose, route and vehicle of infection, we produced a series of caseating granulomas, each of which resembled a particular human lesion. We produced young caseating granulomas in the lung and old encapsulated granulomas with thick fibrous capsules. Erosion of the capsule of such lesions was associated with reactivation TB in the lung as a tuberculous pneumonia characteristic of PPTB. As a consequence, we believe that mice are the best model of caseating granulomas because they can be manipulated to define relevant mechanisms. 

Progressive pulmonary TB in rabbits, mice and guinea pigs appear to be models of the early lesion of PPTB. The disease is not due to increasing numbers of viable bacilli, but to a continuous host response to mycobacterial antigens [37]. After containing the initial infection, these animals develop a low level of MTB in their lungs that remains constant for months until the animals die of progressive pathology. This is characteristic of developing human PPTB.

**Figure 8:**
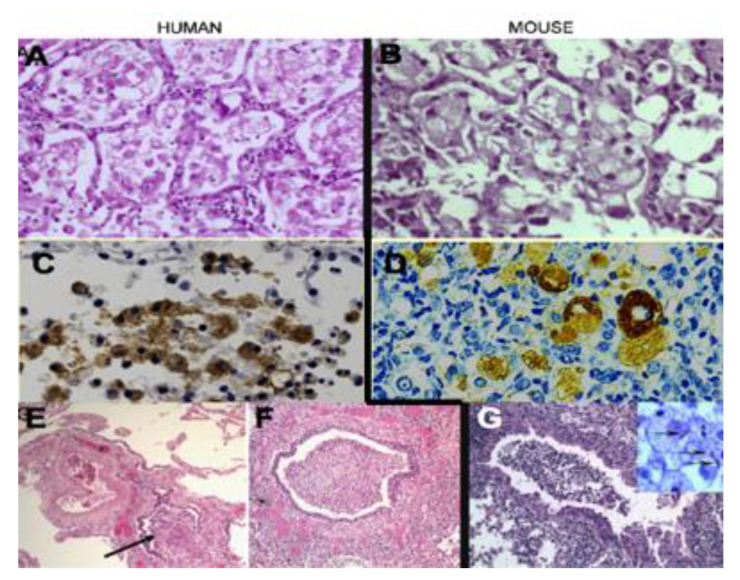
Slowly progressive TB in mice and human PPTB. (**A**) Human early lesion of PPTB (H&E 200×). (**B**) Murine slowly progressive TB (H&E 200×). (**C**) Immunostain of human lesion for MTB antigens (400×). (**D**) Immunostain of mouse lesion for MTB antigens (400×) (**E**,**F**) Endobronchial TB in human lung showing obstruction of the bronchus by inflammatory tissue (arrow) (H&E 40×) (**G**) Endobronchial TB in mouse lung showing similar bronchial obstruction (H&E 100×).

**Figure 9:**
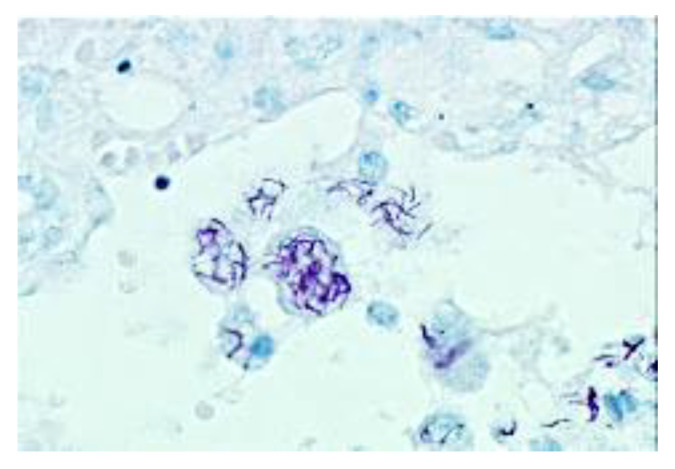
Exacerbation of Chronic TB by TDM. Mice infected i.v. with 10^5^ MTB Erdman develop a chronic interstitial pulmonary infection after several months. This animal was injected i.p. with 100 ug trehalose 6,6′ dimycolate (TDM) on oil and was sacrificed 8 days later. The interstitial infection had been replaced by alveolitis. AFB were found only in alveolar macrophages (AFB stain 1000×). This is an extreme example of infection restricted to alveolar macrophages.

Slowly progressive pulmonary TB in immunocompetent mice has additional correlates with the early lesion of PPTB [7,8,136,137,138]. It is an obstructive pneumonia with sparsely infected foamy alveolar macrophages that slowly accumulate host lipids and secreted mycobacterial antigens in a pattern similar to the human disease, Figure 8. The infection is restricted to alveolar macrophages even after massive enhancement by i.p. injection of cord factor [139], Figure 9. No organisms were found in any other part of the bodies of these animals. The murine infection is maintained by the continuous expression of T_H_1 immunity as evidenced by the demonstration that depleting mice with stationary lung infection of CD4^+^ T cells results in a resumption of MTB growth, as does treatment with an NOS2 inhibition [140]. Stationary lung infection has been shown to be associated with the presence in the lungs of replicating CD4^+^ T cells capable of making IFN-γ in response to MTB antigens. These lesions expand until the animals rapidly develop symptoms and die of pulmonary failure. There is no caseation necrosis, so the lesions do not develop as they do in humans. However, they are models of the early lesion of PPTB.

**Figure 10:**
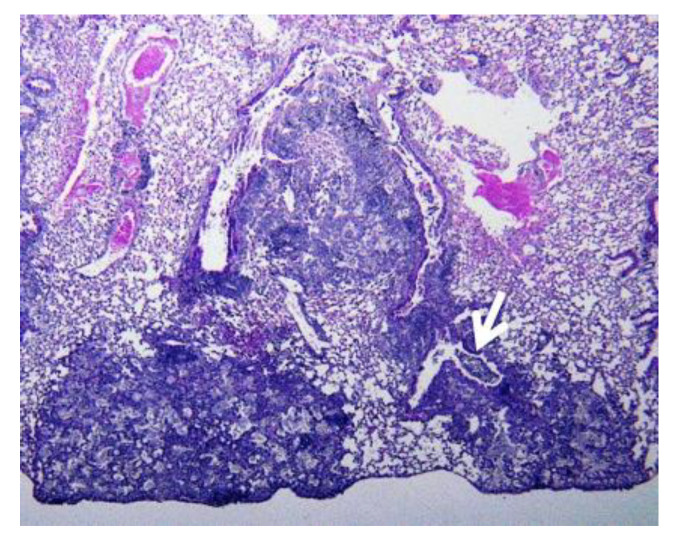
Reactivationå TB in Cornell Model. A/J mice were infected with MTB Erdman by aerosol and treated with INH and PZA for 12 weeks to produce latent infection. This is a reactivation lesion at 200 days. Note the wedge or fan shaped alveolitis and bronchial obstruction with no granulomas that are characteristics of the early lesion of human PPTB. (H&E 4×).

Reactivation TB in mice produced by the Cornell model is an even better model of developing human post-primary TB because it begins like the human disease as sub pleura, wedge shaped lesions of bronchogenic obstructive lipid pneumonia, Figure 10 [131].

TB in non-human primates requires comment. The statement that TB pathology in macaques shows a “striking similarity to classical TB histopathology in humans” refers only to primary granulomas [141,142]. The pneumonia reported in primates resembles a perifocal reaction surrounding a primary TB granuloma, not an early lesion of PPTB [143]. There is no description of bronchogenic spread of tuberculous pneumonia, post-primary granulomas or formation of cavities by dissolution of caseous pneumonia. Finally, non-human primates are reported to produce only weak DTH reactions [144,145]. Since human PPTB is associated with exceedingly strong DTH reactions, this suggests that it might be difficult to induce such reactions in non-human primates. Nevertheless, there is no reason to believe that non-human primates cannot be valuable models of some aspects of TB, but, like all other species, there must be critical examination of what is relevant and what is not.

## 9. Immunity to Primary and Post-Primary TB

It is necessary to remember that MTB is an obligate human parasite that must protect its host and develop means to escape to new hosts. These are the functions of primary TB and PPTB, respectively [7,9,77]. Unlike many infections where strong immunity confers protection, a strong skin test response to tuberculin is a sign of resistance to primary and extra-pulmonary TB, but increased risk of severe disease and death from PPTB [21]. Furthermore, the antigens involved are largely secreted by the organism into its environment [138]. Immunity to primary TB seems adequately explained by the prevailing dogma that T cells produce IFN-γ that activates macrophages to kill MTB or form granulomas to isolate them. ‘Immunity’ to PPTB is different and unique. It can be mediated by any one of a large number of factors that hinder development of the early lesion prior to onset of caseous necrosis. It seems that Dannenberg was correct. The same beneficial DTH reaction that is responsible for activation of macrophages to kill MTB is, with excess bacillary antigen, also responsible for almost all of the tissue damage produced by this disease including granulomas, perifocal reactions, caseation necrosis, liquefaction, and tuberculous pneumonia [71].

While much remains to be learned, we know that ‘protection’ from clinical PPTB can be provided by any of several factors that impede development of the early lesion. The most perplexing factor is the requirement for strong immunity to produce a subclinical early lesion. Weak immune responses due to young or old age, drugs or infections such as HIV increase susceptibility to primary and disseminated TB, but reduce it to PPTB. As discussed earlier, PPTB also employs an array of immunoregulatory functions that serve to maintain both MTB secreted antigens in foamy alveolar macrophages and highly sensitized T cells in close proximity to one another. Perturbation of these functions by vaccines or host-directed therapy have shown promise. The early lesion also requires bronchial obstruction to further isolate it [85]. Relief of obstruction by surgery or regression of an enlarged lymph node may cause its regression [2]. Another factor is that granulomas of PPTB actually surround and stop growth of the early lesions. Medlar examined thousands of post-primary granulomas and found no evidence that any of them grew in size or eroded into anything [49]. Post-primary granulomas begin by surrounding existing foci of caseous pneumonia and then shrink slightly as the caseation ages. This process produces the reverse halo sign on X-ray [119,146].

Finally, an intriguing possibility derives from the observation that the majority of immune dominant epitopes of MTB are hyperconserved meaning that they exhibit less variation than is found in essential genes [147,148,149]. This suggests that the exact structure of hyper conserved epitopes is required for transmission to new hosts. Since there is considerable variation in many aspects of both the hosts and organisms, it seems unlikely that a change in a single epitope among hundreds could alter the immune response sufficiently to abort transmission. An intriguing possibility is that the exact structure of the hyper conserved epitopes is required to keep them sequestered for months in the early lesion of PPTB. If an epitope caused leakage of antigen during this time, it would initiate a surrounding granulomatous reaction that would halt progress toward cavitation and transmission.

## 10. Potential of Advancing Technology

As stated by Douglas Young, Chair of the New Vaccines Working Group of the Stop TB Partnership “we really need to generate a better understanding of human TB and identify which aspects can most usefully be modeled in experimental animals. The route to better animal models is inextricably linked to a better understanding from direct studies of human infection” [127]. However, obtaining informative human tissue for research is a major problem. Peripheral blood and BAL cannot be expected to decipher multiple types of lesions that exist simultaneously a lung. The developmental lesions of PPTB are seldom present in surgical resections because of prior therapy. For practical purposes, autopsies of people with untreated TB are frequently the only source of tissue. Some have thought that autopsies contain only advanced lesions. However, lungs of people who die of TB frequently contain multiple stages of disease including both beginning and advanced lesions. Surgical resections, in contrast, seldom contain the early lesion because there is no ethical reason to resect them.

The numbers of autopsies and thus the availability of such tissue has declined greatly since the end of the preantibiotic era. However, around 4000 people still die of TB every day. If there were a will, surely there would be a way. In addition, with advancing technology, we can now do hundreds of studies on slides from a single block using recent cases or archives that exist in pathology departments around the world [150]. The challenge is to make such tissues available so that TB researchers will be able to relate their results to the actual human disease. O’Garra wrote “Provided that one attempts to relate findings about the immune responses in experimental models back to the immune response occurring during the different stages of human TB, we will be able to use the information to increase our knowledge of this complex disease and to move toward improved diagnosis, prognosis, drug treatment, and vaccination” [128].

## 11. Summary

Research on the pathogenesis of TB today is driven by the paradigm that granulomas are the hallmark of the disease. An extended review of the literature revealed that this is a product of animal studies of the late 20th century that has no support among investigators who actually studied the pathology, radiology and clinical course of untreated human TB. An enduring mystery is how the immune response can simultaneously protect and cause tissue damage. We can understand why people with weak immune responses develop disease, but not why people with the strongest immune responses also have greater risk of clinical disease and death?

As a successful human parasite, MTB has evolved to protect its host and to escape to find new hosts. These are the functions of primary TB and PPTB respectively. This paper integrates clinical, X-ray, pathologic and immunologic data from the pre-antibiotic era with recent studies to present evidence that these two functions require similar, if not identical, immune responses. Primary TB develops when a person is unable to mount a sufficient immune response. PPTB, in contrast, develops most severely in people with the strongest immune responses. MTB has developed means to use our strongest immune response to produce pulmonary cavities from which it can escape to new hosts while, at the same time, maintain a high degree of immunity in all other parts of our bodies. It begins as the early lesion; a prolonged asymptomatic stockpiling of secreted mycobacterial antigens in foamy alveolar macrophages and in close association with highly sensitized T cells in preparation for a massive necrotizing hypersensitivity reaction, the Koch Phenomenon, that produces caseous pneumonia that is either coughed out to form cavities or retained to become the focus of post-primary granulomas and fibrocaseous disease.

The granulomas produced by primary TB and PPTB are morphologically distinct and arise from different types of lesions, but have similar functions. They both form to surround and isolate infectious foci and they both protect against disseminated TB. Once primary TB has induced sufficient resistance to infection, all subsequent infections and spread of infection occur in resistant soil as PPTB. This is a unique disease process that has no known counterparts in other infections and is not fully reproduced in any animal model. It is unique to humans and is responsible for 80% of clinical disease and nearly 100% of transmission to new hosts.

In considering animal models, it is important to recognize that the most important factor in determining the type of disease produced by MTB is prior sensitization to mycobacterial antigens. Once a person or animal has been sensitized, the nature and course of all subsequent infections will be profoundly different. This was well known in the preantibiotic era, but has been largely forgotten. Nevertheless, recent studies have used this principal to produce cavities in rabbits and caseating granulomas in mice [133,135]. Failure to recognize it has led misinterpretation of primary TB in primates as being representative of all human lesions. This is important in developing vaccines and host directed therapies because protection from primary TB and PPTB are mediated by very different mechanisms. Strong immunity to primary TB is associated with greater risk of disease and death from PPTB.

The early lesion of PPTB appears to be its Achilles’ heel. The vast majority of such lesions regress spontaneously in humans. If we knew why, means might be found to make them all regress and thereby stop transmission and drive MTB to extinction. Available evidence suggests a number of factors including failure of bronchial obstruction, premature development of granulomas, weak immune responses and altered macrophage polarization that impede its development. However, much remains to be learned.

We can now identify components of the early lesion in several animal models as the stationary level of infection that persists after the primary peak subsides. These are not perfect models since most animals eventually die of progressive disease whereas most humans resolve the primary infection. Nevertheless, progressive pulmonary TB is not due to increasing numbers of viable bacilli in rabbits, mice and guinea pigs, but is due to a continuous host response to mycobacterial products. “Tuberculosis progresses if the bacillary antigens are continuously produced and regresses if they are destroyed” [37]. This appears to be a common feature of both the human disease and several animal models that could be exploited as a target for vaccines or host-directed therapies.
